# Policy for transitioning childhood-onset growth hormone deficiency from pediatric to adult endocrine care in Belgium

**DOI:** 10.3389/fendo.2024.1459998

**Published:** 2024-09-30

**Authors:** Willem Staels, Jean De Schepper, Marianne Becker, Philippe Lysy, Daniel Klink, Karl Logghe, Marieken den Brinker, Anne Rochtus, Bruno Lapauw, Martine Cools, Orsalia Alexopoulou, Marie Bex, Bernard Corvilain, Laurent Crenier, Christophe De Block, Julian Donckier, Robert Hilbrands, Michel Ponchon, Guy T'Sjoen, Annick Van Den Bruel, Sara Vandewalle, Brigitte Velkeniers

**Affiliations:** ^1^ Division of Pediatric Endocrinology, Department of Pediatrics, Vrije Universiteit Brussel (VUB), Universitair Ziekenhuis Brussel (UZ Brussel), Brussels, Belgium; ^2^ Genetics, Reproduction and Development (GRAD), Vrije Universiteit Brussel (VUB), Brussels, Belgium; ^3^ Division of Pediatric Endocrinology, Department of Pediatrics, Centre Hospitalier de Luxembourg (CHL), Luxembourg, Luxembourg; ^4^ Division of Pediatric Endocrinology and Diabetes, Cliniques Universitaires Saint Luc, Brussels, Belgium; ^5^ Division of Pediatric Endocrinology and Diabetes, ZNA Queen Paola Children’s Hospital Antwerp, Antwerp, Belgium; ^6^ Department of Pediatrics, AZ Delta, Roeselare, Belgium; ^7^ Division of Pediatric Endocrinology, Department of Pediatrics, Universitair Ziekenhuis Antwerpen, Universiteit Antwerpen (UA), Antwerp, Belgium; ^8^ Division of Pediatric Endocrinology, Department of Pediatrics, Universitair Ziekenhuis Leuven, Leuven, Belgium; ^9^ Department of Endocrinology, Universitair Ziekenhuis Gent, Ghent, Belgium; ^10^ Department of Paediatrics, Division of Paediatric Endocrinology, Ghent University Hospital, Ghent University, Ghent, Belgium; ^11^ Department of Internal Medicine and Paediatrics, Ghent University, Ghent, Belgium; ^12^ Department of Endocrinology, Cliniques Universitaires Saint Luc, Brussels, Belgium; ^13^ Department of Endocrinology, UZ Leuven (Universitaire Ziekenhuizen Leuven), Leuven, Belgium; ^14^ Department of Endocrinology, Hôpital Erasme, Université Libre de Bruxelles, Brussels, Belgium; ^15^ Department of Endocrinology and Diabetes, Universitair Ziekenhuis Antwerpen (UZA), Universiteit Antwerpen (UA), Antwerp, Belgium; ^16^ Department of Endocrinology, Université Catholique de Louvain CHU UCL Namur (Site Godinne), Yvoir, Belgium; ^17^ Department of Diabetes and Endocrinology, Vrije Universiteit Brussel (VUB), Universitair Ziekenhuis Brussel (UZ Brussel), Brussels, Belgium; ^18^ Department of Endocrinology, Cliniques Saint Jean, Brussels, Belgium; ^19^ Department of Endocrinology, AZ Sint Jan Brugge AV, Brugge, Belgium

**Keywords:** growth hormone deficiency (GHD), growth hormone therapy, transition, growth hormone stimulation tests, policy for transition care

## Abstract

Growth hormone (GH) deficiency (GHD) in children and adolescents can vary in severity and origin, with GH replacement therapy proving effective in achieving genetic target height. Optimal outcomes are seen in those treated early and with higher doses. As patients approach adult height, priorities shift towards optimizing metabolic effects, maintaining body composition, and enhancing bone mass and muscle strength. Transitioning from pediatric to adult care presents challenges, including accurately identifying candidates for continued GH therapy, reevaluating persistent GHD, and preventing treatment discontinuation. Assessing readiness for transition and self-management skills is crucial. This Policy and Practice Review provides a comprehensive overview of current policies, regulations, and guidelines pertinent to managing GHD transition in Belgium. We integrate perspectives from national academic and nonacademic clinical stakeholders in pediatric and adult endocrine care to provide an updated policy framework. This framework underscores the importance of sustained GH therapy during transition, particularly for individuals with persistent GHD, with the goal of optimizing practices and improving outcomes during this critical period.

## 1 Introduction

Growth hormone (GH) deficiency (GHD), when diagnosed during childhood or adolescence, can manifest as an isolated or a combined pituitary hormone deficiency, and it can vary in severity, ranging from severe (often defined as a peak GH following a stimulation test of < 3 ng/mL) to partial GHD (often defined as a peak GH of 3-7 ng/mL) (1). GHD may arise from idiopathic, genetic, or acquired origins, yet in each scenario, GH replacement therapy has demonstrated its efficacy in facilitating the attainment of an adult height within the genetic target height. Notably, the most favorable growth outcomes have been documented in patients with combined GHD, particularly those who started treatment at the youngest age and/or received the highest GH doses (2–5).

As individuals approach their near-adult height, the focus of GH therapy shifts from optimizing growth towards optimizing the metabolic effects of GH, primarily directed at maintaining a healthy body composition and achieving optimal peak bone mass and muscle strength in early adulthood and a transfer from pediatric to adult care becomes imperative (6–9). However, transitioning adolescents or young adults with GHD from pediatric to adult endocrinology departments poses multifaceted challenges, including medical, psychosocial, and organizational aspects (10–13). Key clinical challenges include accurately identifying candidates suitable for seamless continuation of GH treatment or reevaluation for persistent GHD, along with precise GHD diagnosis for those who require retesting (14). Additionally, strategies to mitigate the risk of follow-up loss and/or discontinuation of GH treatment need to be developed, particularly for adolescents with isolated GHD, a group that is more vulnerable to early discontinuation of medical surveillance (15). Hence, assessing readiness for transition, disease comprehension, and self-management skills are essential before transitioning patients to adult care (16).

The prevailing guidelines set forth by scientific societies for the transition care of adolescents with GHD are based on a combination of clinical expert consensus and the growing body of evidence from clinical studies, showing the advantages of continued GH therapy during the transition phase, especially for those with persistent GHD (17, 18). Differences in national policies regarding the testing of persistent GHD and the continuation of GH treatment, in addition to the observation of non-uniform GH dosing, along with the significant drop-out rates after departing pediatric services, have driven the need to update the guidelines governing the management of GH therapy during transition (19–23).

## 2 Assessment and discussion of policy options and implications

### 2.1 Why is a Belgian policy needed?

In this Belgian policy, we adapt international guidelines to our specific national context to standardize and optimize GHD management during the transition from pediatric to adult endocrine care. Key considerations included the expanding gene panel use for diagnosing GHD, addressing disparities in clinical practice among Belgian hospitals, and the recent changes in GH reimbursement criteria for GHD during the transition phase. Adherence to national policy can result in improved cost-effectiveness. However, the pivotal step lies in adopting these proposed guidelines by national insurance policies, ensuring their effective integration into the Belgian healthcare framework.

### 2.2 How were the Belgian guidelines established?

A writing committee comprised of national experts from Belgian university and non-university hospitals was established. First, two in-person meetings were held between several authors (WS, JDS, MB, PL, DK, KL, CDB, MB, BV) to review and discuss current evidence. The international guidelines and recent publications formed the basis for practical recommendations adapted to the Belgian context (12, 28-31). Next, the other co-authors were consulted through consecutive e-mail rounds. Finally, the guidelines were endorsed by the Belgian and Luxemburgish Society for Pediatric Endocrinology and Diabetology (BELSPEED), the Belgian Endocrine Society (BES), and the Flemish Network for Rare Diseases (VNZZ).

### 2.3 What is the current management of GHD during childhood and transition in Belgium?

In Belgium, GH therapy for children with GHD is managed by pediatric endocrinologists, who convene monthly for peer-review of diagnostic assessments related to GHD, coordinated by BELSPEED. These assessments encompass clinical, auxological, hormonal, brain imaging, and bone age evaluations. The diagnostic criteria include (a) growth velocity below the 25^th^ percentile (in prepubertal patients), (b) delayed bone age maturation, (c) serum IGF1 level below the mean value for the corresponding age/bone age, and (d) GH peak < 7 µg/L (since 2009) elicited during two GH stimulation tests. Notably, at least one of these tests should be sex steroid primed in girls aged above 9 years and boys aged above 10 years, who have not yet reached an advanced pubertal stage (Tanner B3 in girls, testicular volume ≥ 10 ml in boys). Furthermore, patients diagnosed with GHD routinely undergo a brain MRI. Annually, Belgium registers 50 to 70 cases of GHD in children.

GH dosing is body weight-based at 25-30 µg/kg/day until near-adult height. GHD diagnosis is reevaluated by the treating pediatric endocrinologist after one year, juxtaposing the growth response with national references (24). Retesting for persistent GHD during mid-puberty is not customary in Belgium except when the need to continue the GH treatment is questioned by the patient, compliance is poor, or alternative diagnoses are considered. Typically, GH treatment follows the above pediatric dosing regimen as long as the growth velocity exceeds 2 cm/year and/or bone age readings show open growth plates. When these criteria are no longer met, a short-term suspension of GH therapy is done for all patients with idiopathic GHD and most patients with organic GHD. In 2003, we reported that 33% of patients with isolated idiopathic GHD exhibited a transient form when re-evaluated based on the current GHD criteria after reaching adult height (25). Unfortunately, recent national data on retesting outcomes are not available.

In most Belgian centers, an insulin tolerance test (ITT) is performed to evaluate persistent GHD. The testing is, in general, performed between 1 and 6 months after stopping GH therapy, but different cut-offs of peak GH value (varying between < 3 and < 7 µg/L) are used to diagnose persistent GHD. Clinical practice ensuring continued GH treatment beyond pediatric care varies among centers. In some, the transfer to adult endocrine care occurs upon reaching adult height, while in others a transition near the age of 18 years is more common. The handover of care in most pediatric centers is realized through a referral letter to the adult endocrinologist, while some centers arrange joint consultations with pediatric and adult care providers.

### 2.4 How should the transitioning of adolescents treated for GHD be organized?

The initiation of the transfer process to adult endocrine care should be guided by the adolescent’s readiness, even if final height is not yet attained. Pediatric endocrinologists should strive to equip adolescents with the skills to proficiently manage GHD without relying on parental supervision. Employing specific questionnaires, checklists, or tool kits can aid in assessing readiness and identifying knowledge gaps before transitioning to adult care. Notable examples of validated checklists include the Transition Readiness Assessment Questionnaire (TRAQ) and the Transition Readiness and Appropriateness Measure (TRAM) (16, 26).

The seamless transition of care necessitates close collaboration between pediatric and adult endocrinologists, facilitated by joint consultations. Due to the increased need for social support, lower general well-being, and the increased risk of fertility issues in adolescents with multiple pituitary hormone deficiencies (MPHD), transitioning to a multidisciplinary clinic proves most appropriate (27, 28). Such multidisciplinary transition clinics ideally encompass access to psychologists and fertility counselors, including gynecologists and andrologists (29). A dedicated adult clinic case manager can streamline patient transitions and reduce follow-up gaps.

In Belgium, established multidisciplinary transition services for GHD patients remain scarce. These clinics must establish therapeutic education programs as patients with persistent GHD often require comprehensive and repeated explanations of the benefits of continued GH treatment after having reached adult height. These include improved body composition, bone mineralization, vascular health (including serum lipids), and quality of life (30). Implementing case managers could significantly improve transition care by coordinating care and tailoring support according to individual patient needs (31). Unfortunately, case managers are unavailable in Belgium, and governmental or private healthcare insurance do not foresee their reimbursement. Particularly for patients with cognitive challenges, commonly observed in those with GHD after brain irradiation or injury, the additional guidance by case managers is beneficial. To achieve these objectives, facilitating reimbursement for transition clinics focused on endocrine conditions and organizing dedicated personnel education is paramount.

### 2.5 When and why should GH therapy be interrupted or continued?

The critical moment to decide on continuing GH treatment is at the end of skeletal growth. The conclusion of longitudinal growth is typically marked by a decline in height velocity to below 2 cm/year, which can, in case of doubt, be radiologically confirmed by near-final bone maturation, predictive of about 99% of residual skeletal growth, i.e., a Greulich and Pyle scored bone age of 15 years in girls and 17 years in boys.

The estimates of persistence of childhood-diagnosed GHD into adulthood are variable, ranging from 12.5% in isolated GHD to 89% in MPHD (20, 32, 33). Key determinants of GHD persistence encompass GHD etiology and severity, age at diagnosis, and the presence of concurrent pituitary hormone deficiencies (33–35). When the likelihood of persistent GHD is high, GH therapy should not be interrupted. Such interruption periods often remain asymptomatic, and some patients, especially males, are reluctant to resume GH therapy afterwards (20). Though variable, adverse changes in body composition, such as an increase in body fat and a decrease in lean mass, alongside reduced quality of life, can arise as early as 6 months after GH interruption (36, 37). Prolonged interruptions can result in dyslipidemia and increased carotid artery intima-media thickness, which compromises cardiometabolic health (37–43), a decline in muscle force, and lower quality of life (44).

**Figure 1:**
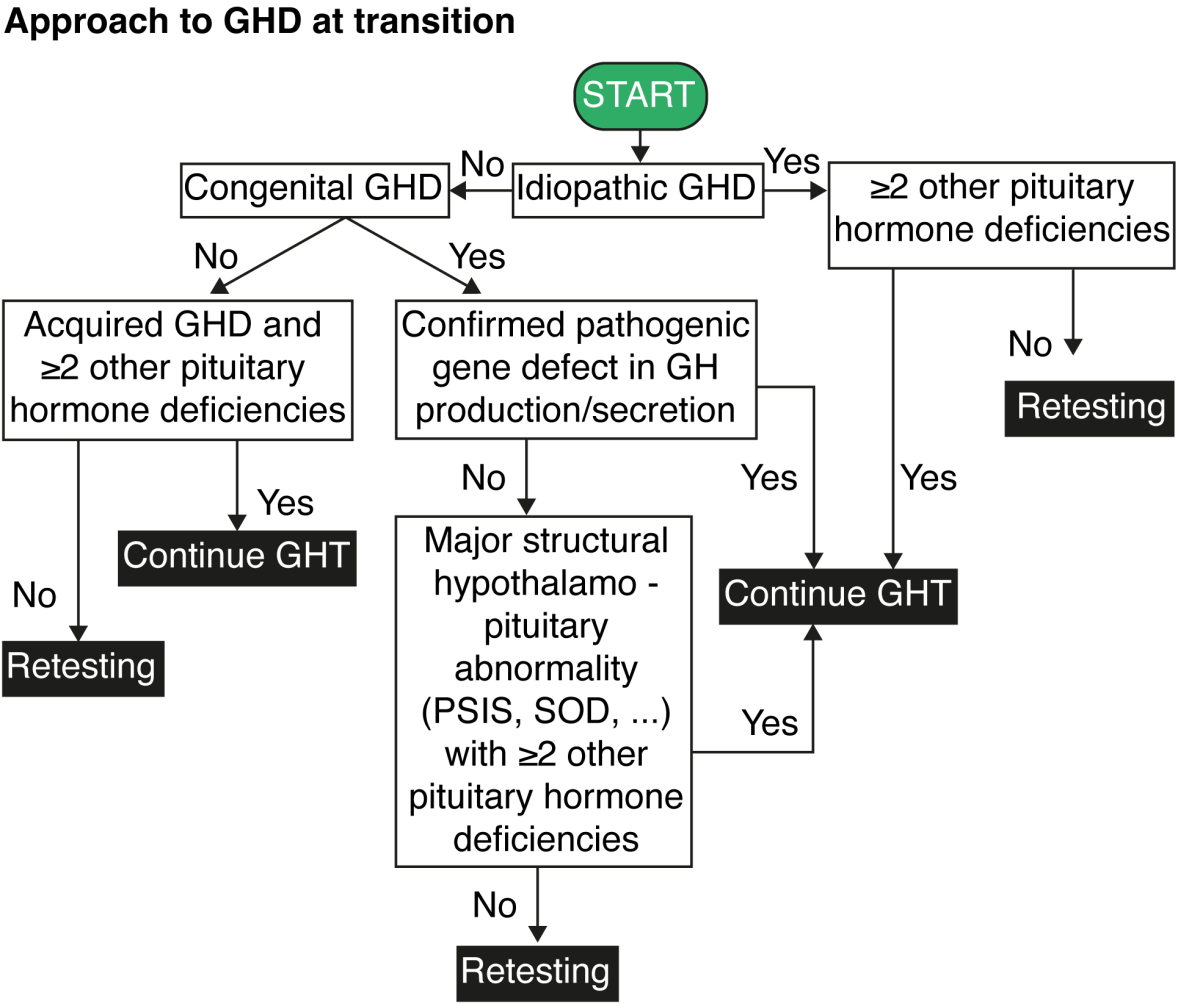
Flowchart for the approach to growth hormone deficiency at transition. GH, growth hormone; GHD, growth hormone deficiency; GHT, growth hormone therapy; PSIS, pituitary stalk interruption syndrome; SOD, septo-optic dysplasia.

**Figure 1** presents a decision flowchart outlining the considerations for either adapting the GH dose and continuing or temporarily interrupting treatment to assess for persistent GHD.

### 2.6 Which patients should continue GH treatment?

The decision regarding the continuation of GH therapy relies on several key factors, encompassing five distinct patient profiles.

#### 2.6.1 Confirmed gene defects responsible for impaired GH secretion

Continuation of GH therapy is warranted for individuals with pathogenic variants in genes governing pituitary gland development, GH synthesis, or release, encompassing both isolated GHD and MPHD cases. Confirmation of genetic GHD forms requires independent genetic analysis, irrespective of affected relatives or parental consanguinity. In the absence of a molecular diagnosis at transition, clinical cues can guide targeted gene analyses.

For early-onset severe isolated GHD with a small to normal-sized pituitary on MRI, variants in *GH-1* and *GHRHR* should be investigated. *POU1F1* and *PROP-1* sequencing is recommended for combined GHD without pituitary anomalies, often accompanied by TSH and PRL deficiencies (34, 45). A short neck with restricted rotation, with or without sensorineural hearing loss, warrants *LHX3* mutation analysis. Cerebral malformations such as hypoplastic corpus callosum or Chiari malformation suggest *LHX4* gene variants. Bilateral microphthalmia points to variants in the *SOX2*, *OTX2*, or *RAX* genes. Routine testing for variants in these genes is currently available at various medical genetic centers in Belgium.

#### 2.6.2 Major congenital midline abnormalities

Adolescents with radiologically confirmed substantial congenital midline defects affecting the hypothalamic-pituitary region, such as pituitary stalk interruption syndrome (PSIS) or septo-optic dysplasia (SOD) should continue GH therapy if they present with an additional deficiency of ≥ 2 other pituitary hormones. In children with congenital cerebral midline abnormalities, like optic nerve hypoplasia/SOD or PSIS, particularly when diagnosed in infancy, GHD tends to persist, especially in cases with MPHD (32).

#### 2.6.3 Tumor-related GHD

GH therapy should be sustained for individuals who have undergone surgery and/or high-dose irradiation for intra/supra-sellar tumors, leading to MPHD. In young adults with childhood-onset GHD and MPHD due to brain tumors, the persistence of GHD is nearly 100% (20, 32, 46).

#### 2.6.4 Acquired non-tumoral GHD

In cases of acquired non-tumoral GHD resulting from conditions such as severe brain trauma, lymphocytic hypophysitis, sarcoidosis, or Langerhans cell histiocytosis, continuation of GH therapy is indicated, particularly in MPHD (47, 48).

#### 2.6.5 Idiopathic GHD

Adolescents with idiopathic GHD exhibiting ≥2 other pituitary deficiencies should continue GH treatment. Although MPHD is infrequent in children with idiopathic GHD, a persistence of the GHD is observed into adulthood in such cases (25, 49).

### 2.7 When, where, and how to conduct retesting for GHD?

Retesting for GHD should be done in isolated GHD or GHD combined with only one other pituitary deficiency, particularly in the following scenarios:

#### 2.7.1 Normal genetic testing and normal brain imaging, considered idiopathic GHD

Most adolescents with idiopathic GHD, either isolated or combined with only one other pituitary deficiency, exhibit normal GH reserves upon testing in adulthood (35, 50–52).

#### 2.7.2 Isolated anterior pituitary hypoplasia or isolated ectopic posterior pituitary findings on brain MRI

GHD in children with isolated ectopic posterior pituitary or PSIS without other pituitary hormone deficiency is not invariably permanent, necessitating retesting to avoid unnecessary treatment (21, 53–55). Normal GH peaks upon retesting have also been noted in adolescents with a small pituitary (56).

#### 2.7.3 GHD diagnosis after brain irradiation for a tumor outside the hypothalamic-pituitary region or hematologic malignancy

Up to 45% of children initially diagnosed with GHD after brain irradiation for a distant brain tumor, mainly medulloblastoma, no longer show GHD upon retesting in adulthood (57, 58). The cranial irradiation dose received during childhood determines the GH peak upon retesting in young adulthood (59).

#### 2.7.4 GHD diagnosed after a traumatic brain injury

Only a third of children treated for GHD after traumatic brain injury demonstrated confirmed GHD in young adulthood (60).

#### 2.7.5 GHD related to Prader-Willi syndrome

Merely a fifth of GH-treated adolescents with Prader-Willi syndrome were GHD upon reaching adult height (61).

Retesting is ideally initiated by the pediatric endocrinologist upon approaching near-adult height (defined as a height gain ≤ 2 cm in the preceding year) and performed in the pediatric clinic. A wash-out period of 3 months is recommended to mitigate false positive results in GH stimulation tests. This recovery period allows for restoring normal GH secretion physiology, optimization of other concurrent hormone therapies, and cessation of estrogen-containing oral contraceptives. These measures ensure accurate interpretation of serum IGF1 and peak GH values during the stimulation test (32, 62–64). Notably, optimizing thyroid hormone levels is essential for reliable interpretation of the GH stimulation test. Sex steroid replacement therapy, like estrogens or androgens, should not be discontinued before retesting (65). However, in patients previously diagnosed with gonadotropin deficiency, whether partial at initial testing or linked to a family history of delayed puberty, stopping testosterone or estrogens for 3 months before GH retesting is recommended. This halt should be used to confirm permanent gonadotropin deficiency and, when confirmed, recommence sex steroid treatment for at least 3 months before GH retesting.

### 2.8 How to confirm the diagnosis of persistent GHD in adulthood?

We recommend that the diagnosis of GHD is confirmed through a combination of a GH stimulation test and measurement of serum IGF1 levels following the discontinuation of GH treatment. We do not recommend omitting the need for a GH stimulation test when serum IGF1 levels are either < -2 SD (indicative of GHD) or normal (dismissing GHD) after halting GH therapy (17) as opposed to others (32, 46, 50). Several studies demonstrate that a low serum IGF1 level alone, or even combined with low serum IGFBP3, does not confirm GHD in adolescents or young adults (32, 46, 50, 66).

#### 2.8.1 Pitfalls for relying on IGF1 levels alone in diagnosing GHD

First, interpreting IGF1 results can be complicated due to the variation among different IGF1 assays, stemming from differences in antibody specificity and pre-analytical sample preparation strategies to eliminate binding protein interferences (67–69). Serum IGF1 is best measured using a total IGF1 assay employing the NIBSC (02/254) standard, which offers reliable age-dependent reference values (70). Secondly, there is a major fluctuation in IGF-1 levels. After extended GH treatment, it might take 6-12 months for serum IGF-1 levels to revert to their endogenous production levels (32). Time since GH cessation, pubertal development status, body adiposity, and associated conditions can all complicate serum IGF1 interpretation. Factors such as obesity, insulin resistance, delayed gonadal development, chronic inflammation, low-calorie intake, and use of estrogens (such as oral contraceptives) can lead to varying serum IGF1 levels. Up to 50% of adolescents initially diagnosed with idiopathic GHD during childhood exhibited low serum IGF1 levels after GH treatment cessation but were later diagnosed as having transient GHD (34, 71). Conversely, around 25% of adolescents with confirmed GHD during transition had normal serum IGF1 values (generally below the mean) (21, 32, 50, 71). Therefore, a serum IGF1 below -2 SD can be considered suggestive but not diagnostic for GHD, whereas a normal IGF-1 level can be considered suggestive of normal GH production but does not exclude persistent GHD.

#### 2.8.2 The need for GH provocation tests in diagnosing GHD

To reliably ascertain the persistence of GHD, the primary criterion is the failure to achieve a specific peak GH cut-off level during a GH stimulation test. Based on Belgium’s experience with the insulin tolerance test (ITT) and glucagon testing (GST), these two tests are preferred during the transition phase. The ITT, unless contraindicated, e.g., in the case of epilepsy, is considered the primary choice (72). In cases where epilepsy or other contraindications preclude the ITT, a glucagon stimulation test (GST) is an alternative.

**Figure 2:**
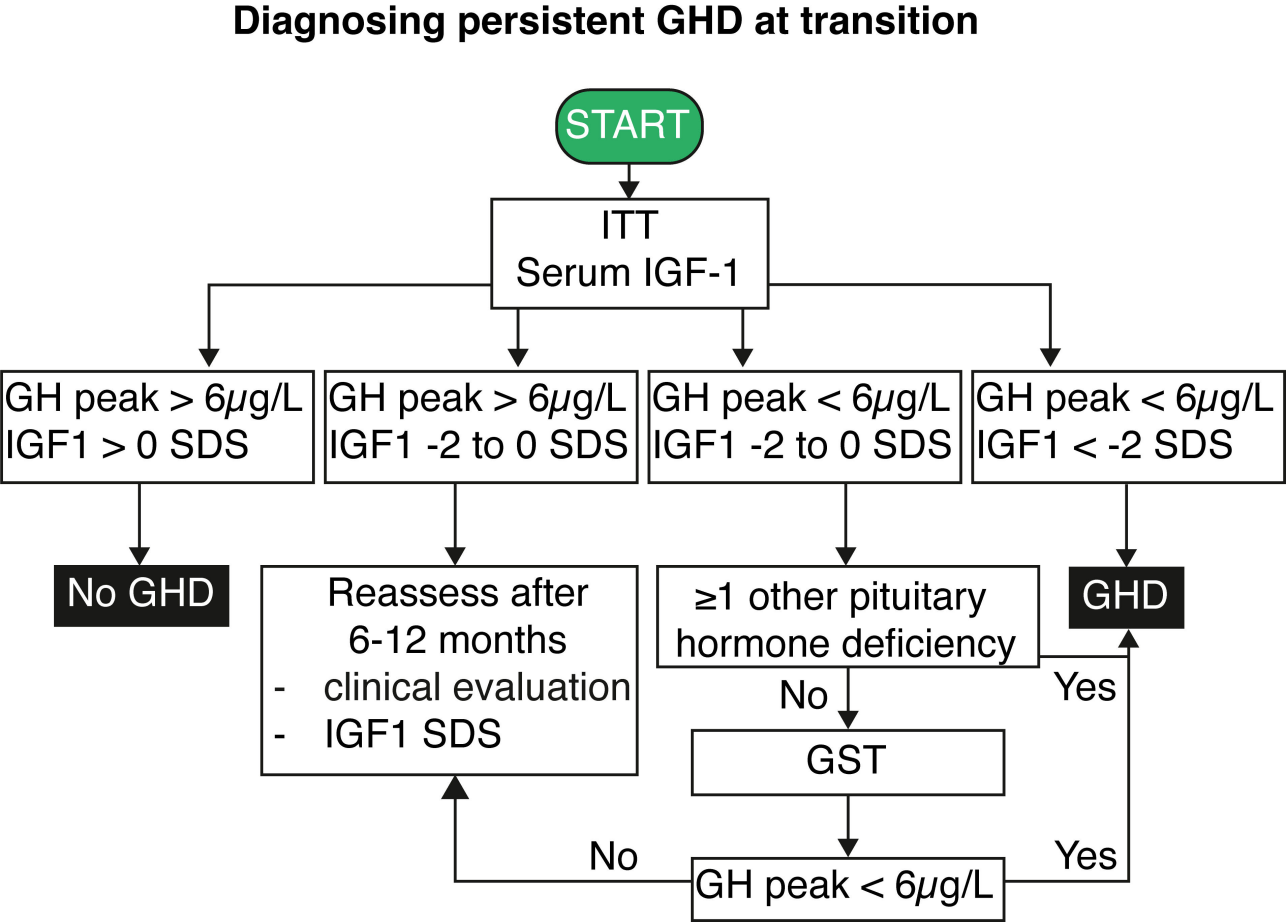
Flowchart for diagnosing persistent growth hormone deficiency at transition. IGF1, insulin-like growth factor 1; GH, growth hormone; GHD, growth hormone deficiency; GST, growth hormone stimulation test; ITT, insulin tolerance test; SDS, standard deviation score.

An algorithm detailing the diagnostic process for persistent GHD based on serum IGF1 and ITT results is shown in **Figure 2**. The criteria encompass a combination of serum IGF1 levels and peak GH responses to determine the presence of GHD. Additional considerations, such as pituitary hormone deficiencies and individual medical histories, guide further assessments and evaluations for accurate diagnosis and management. Briefly, GHD can be excluded when IGF1 levels are above the mean, and a GH peak above 6 µg/L is obtained at the ITT. The diagnosis of persistent GHD can be made when serum IGF1 levels are below the - 2 SD limit and a peak GH below 6 µg/L after insulin (or below < 3 µg/L after intramuscular glucagon administration) stimulation is found. In those adolescents with MPHD and low-normal serum IGF1 levels (between -2 and 0 SD), the presence of GH peak < 6 µg/L at the ITT is sufficient to confirm persistent GHD (73). On the other hand, adolescents with low-normal serum IGF1 (near the age-related lower limit or between -2 and 0 SD) and a peak GH < 6 µg/L but no additional or only one associated pituitary hormone deficiency require a second GH stimulation test with another secretagogue (preferentially glucagon) given the known variability of peak GH values at ITT/GST testing and basal serum IGF1 levels. If the second GH stimulation test yields a GH peak response above the threshold, irrespective of the serum IGF1 level, GHD is unlikely.

However, adolescents meeting the criteria of unlikely persistent GHD, especially those with PSIS or brain irradiation, should be reassessed after 6 to 12 months. Minimum yearly measurements of IGF1, PRL, cortisol, DHEAS, LH, FSH, testosterone or estradiol, FT4, and TSH are needed in brain-irradiated children since they are at risk to develop hypopituitarism over time (55, 58, 74, 75).

### 2.9 How to perform the GH stimulation tests in clinical practice?

GH stimulation tests are best performed approximately three months after GH treatment cessation. They should be performed when the patient feels well, has not performed excessive physical activity in the last 3 days, and always after an overnight fast. Adequate supplementation of other pituitary hormones is key in patients with GHD before testing GH secretion. A comprehensive evaluation of adrenal, thyroid, and gonadal status is crucial for accurate GH stimulation test interpretation. Therefore, prior measurement of morning basal cortisol, ACTH, DHEAS, LH, FSH, testosterone (males), estradiol (females), FT4, and TSH levels, in addition to serum IGF1, is essential (76). Of note, central hypocortisolism should be tested at transition as it develops in up to 44% of young adults with idiopathic GHD (74, 77, 78). Furthermore, the enhanced conversion of cortisone to cortisol during the GH-deficient state may increase basal cortisol levels at retesting.

The ITT should be the first choice and performed using an insulin dose of 0.1 U/kg if BMI ≤ 30 kg/m^2^ or 0.2 U/kg if BMI > 30 kg/m² and sampling at -30, 0, 30, 60, 90, and 120 minutes to determine glucose, growth hormone, and cortisol responses. The -30-minute sample should be taken immediately after intravenous line placement, as in some subjects, the GH response after hypoglycemia is weak, but line placement elicits satisfactory GH secretion (79). A glucose level below 40 mg/dl or a drop of at least 50% of the basal glucose level must be obtained after insulin injection to consider the GH results reliable.

When ITT is contraindicated, the GST can be done using a fixed intramuscular dose of 1 mg (1.5 mg when BMI > 30 kg/m²). Intramuscular stimulation is suggested to be more reliable and effective in releasing GH (86). No specific change in serum glucose after glucagon is needed, but blood sampling for GH measurements is needed at -30, 0, 90, 120, 150, and 180 minutes (80). Sampling at 240 minutes during GST is not needed (81, 82).

### 2.10 How to interpret the insulin tolerance and glucagon stimulation test?

A peak GH value < 6 µg/L following an ITT or GST is considered diagnostic for GHD during the transition phase (50, 56, 83, 84). This cutoff lies between the GH peak of 7 µg/L used in childhood and the 3 µg/L cut-off in adulthood and accords with the physiological decrease in GH secretion after the pubertal growth spurt. In cases of severe obesity (BMI > 30 kg/m²), a lower peak GH cut-off value should be considered (17, 85). For the ITT, the cortisol response above 15 µg/dl is considered sufficient, whereas for the GST, even a lower cut-off of 11 µg/dl can be accepted, at least when the current automated immunochemiluminometric assays are used (86). The choice of GH assay should prioritize chemiluminescence immunoassays for the 22KDa GH form using the second International standard IRP 98/574 and achieve a coefficient of variation < 20% at the lower quantifiable limit. Regular national comparisons among different centers using different GH assays are essential for accurate interpretation.

### 2.11 How should GH treatment be given?

When persistent GHD is diagnosed at retesting, GH treatment should be resumed promptly, even without overt GHD-related complaints, as prolonged interruption of GH therapy can reduce the motivation to resume treatment (20, 87). In adolescents with fused epiphyseal growth plates, pediatric GH doses should be avoided to prevent acromegalic manifestations or fluid retention. To recommence GH treatment within the 3 to 6-month window after an interruption, it is advisable to start with half of the GH dosage administered before the interruption (88, 89). Typically, this dose will be approximately 12.5 - 15 µg/kg/day for most adolescents. Nonetheless, a commencing treatment dose should not exceed 1.2 mg/day (90). When the patient has been without GH replacement for over 6 months, starting at a total daily GH dose of 0.6 to 0.8 mg is more suitable to prevent peripheral edema, arthralgia, myalgia, and paresthesia, related to GH-induced fluid retention (89). The use of disposable pens, although requiring refrigeration, is judged as the most user-friendly device by adolescents and active young adults and can be a suitable choice for continued GH therapy. Administering GH in the evening remains the preferred timing.

Titration of the GH dose can be done after one to two months and, subsequently, every 6 months, aimed at achieving serum IGF1 levels within the appropriate age-related range. Dose adjustments of 0.1 to 0.2 mg/day can be considered when clinical assessments and serum IGF1 levels indicate either over- or undertreatment. The goal is to maintain the IGF1 level around the mean for the given age or, at least, within -2 SD and +2SD if SD reference values for age and gender are available. IGF1 assessments should best be performed at least 6 weeks after a dosage change. Generally, women will receive higher doses than men when titrating GH doses to serum IGF1 levels around the mean. A maximum GH maintenance dose of 2 mg should not be exceeded. This upper limit is rarely needed, even in women taking estrogen-containing contraceptives.

### 2.12 How should GH treatment be monitored, and for how long?

Regular six-monthly visits are recommended, particularly during the first years of transition, due to the substantial risk of loss to follow-up. During these visits, blood pressure, body weight, and waist circumference should be recorded. Beyond the oversight of serum IGF1 concentrations, these visits serve to track potential adverse effects, such as fluid retention, hyperglycemia, arthralgias, and paresthesia. Additionally, they allow assessing underdosing or non-compliance, indicated by increasing waist circumference, reduced vigor, generalized fatigue, or decreased overall well-being. Disease-specific quality-of-life assessment questionnaires can be used to screen for changes in well-being related to GHD. However, whether they should be used to adapt GH dosing remains unclear. After 3-5 years of treatment and/or at the age of 25 years, bone densitometry by DXA is advised. The initial evaluation of the bone mineral density at the lumbar spine and hip is best performed at the adult clinic. This approach ensures reliable longitudinal assessments since DXA techniques may differ between hospitals. Furthermore, regular brain MRI follow-up in patients harboring residual post-surgical brain tumor remnants should be coordinated with oncologists and/or neurosurgeons.

Continuing GH therapy beyond adult height is most beneficial in young adults with more severe clinical and hormonal abnormalities. In cases without discernible clinical benefits after one year of treatment, discontinuing GH therapy may be considered. However, a potential decline in lipid profile, bone mass, muscle mass or strength, and exercise capacity should factor into the decision to resume GH therapy after one year of interruption. If GH treatment is continued, yearly assessments of fasting glucose, total cholesterol, HDL cholesterol, LDL cholesterol, triglycerides, IGF1, FT4, and cortisol are advised. Regular IGF1 monitoring is essential as patients may need higher GH doses over time due to the waning response associated with aging and shifts in body fat composition. Changes from oral to transdermal estrogen replacement or starting an oral estrogen-progesterone combination as contraception could also influence GH dosing.

Around the age of 25 years, when peak bone mass and maximal muscle strength are typically reached, the need for ongoing GH treatment should be reevaluated in those adults with isolated GHD, especially when bone mineral content is within normal limits or when compliance is low. In those patients who continued GH treatment after obtaining final height, the continuation of GH or retesting for persistent GHD should be considered on a case-by-case basis. In these cases, we recommend following international clinical guidelines established for adult-onset GHD treatment (91).

### 2.13 What about long-acting growth hormone?

Long-acting growth hormone (LAGH) preparations for weekly injections have recently become available for treating GHD in several countries. In Belgium, only somatogron is reimbursed for treating GHD in children aged three to 18 years. Weekly LAGH injections are as effective as daily GH injections in promoting growth. While long-term outcome data are not yet available, short-term data indicate a similar safety profile and suggest a lower treatment burden, making LAGH potentially more attractive to children and parents (92, 93). Young children expected to be on GH therapy for many years, children with needle phobia, and children starting self-injections might be good candidates for LAGH (94). However, safety concerns remain, particularly for children with severe GHD experiencing hypoglycemia, cancer predisposition syndromes, GHD after cancer therapy, or severe forms of metabolic syndrome. These children may not be suitable candidates for LAGH (95).

Some safety concerns stem from maintaining supraphysiological GH levels during the day, non-physiological tissue distribution, and different temporal relationships between peak GH and IGF1 with LAGH. While mean serum IGF1 concentrations are comparable between daily and weekly injections, they are higher in the first days after the LAGH injection and might be lower immediately before the subsequent injection compared to profiles observed during standard therapy. Calculators for monitoring serum IGF1 levels in clinical practice were developed for each LAGH preparation but need further evaluation in adolescents and young adults. Additionally, the effects of LAGH during puberty on linear growth, bone, and glucose metabolism still need further study. It is unclear if concomitant thyroxine or hydrocortisone therapies require dose adjustments when switching to LAGH. An international global registry project (GloBE-Reg) was initiated to collect “real-world” safety and efficacy data in children receiving somatogron and daily GH preparation.

LAGH is not yet approved for treating adult GHD in Belgium, but transitioning from adolescents on LAGH to adult care is an opportunity for initiating LAGH therapy in selected adult patients. In adults with childhood- as well as adult-onset GHD, clinical trials have documented a similar reduction in body fat and increase in muscle mass, as well as better treatment adherence compared to daily injections. However, there are no separate data on young adults (96) or young adults transitioning from pediatric daily GH dosing to LAGH. Clinical experience with LAGH in adults is too limited to formulate guidelines on transition (97). A longer LAGH treatment interruption (up to 6 months) could be considered before retesting for GHD at transition.

Most experience with LAGH in adults comes from clinical trials with somapacitan. Based on these trials, a starting dose of 1.5 mg/week (2mg/week for women on oral estrogens) of somapacitan has been proposed after interruption of GH therapy or when initiating GH therapy in young adults, followed by dose adjustments in steps of 0.5 to 1.5 mg at 2-4 weeks intervals (98). Serum IGF1 measurements three days after somapacitan injections can be used to titrate the LAGH dose. In young adults (up to 25 years), serum IGF1 levels are targeted at the upper end of the normal range. Higher GH dosing might improve abdominal fat accumulation but may cause myalgia and insulin resistance (99). The worsening of insulin sensitivity is a concern, especially in those with risk factors for developing diabetes mellitus (e.g., obesity or a family history of diabetes). However, this negative effect of glucose metabolism might be counterbalanced by favorable body composition changes in the long term (100). The effect of LAGH on cardiovascular risk factors and endothelial function needs further study.

## 3 Actionable recommendations

The actionable recommendations are presented in two flow charts: the first outlines the approach to GHD at transition (**Figure 1**), and the second details the process for diagnosing persistent GHD at transition (**Figure 2**). **Table 1** summarizes the key considerations for transitioning GHD from pediatric to adult clinics.

**Table 1:** Overview of important considerations.

Considerations	Details
1.	Treatment with adjusted GH dose (usually about half of the pediatric dose) recommended for adolescents and young adults with persistent GHD at least up to age 25.
2.	Evaluate the need for continued GH treatment when longitudinal growth slows (< 2 cm/year).
3.	Retest for persistent GHD after 3 months without GH treatment.
4.	First-line GH provocation test: Insulin Tolerance Test (ITT). Alternatively, use Glucagon Stimulation Test (GST) if ITT contraindicated.
5.	The cut-off IGF1 value is an age-dependent z-score below -2 (or below 100 µg/L when no z-scores are reported).
6.	GH peak cut-off: < 6 µg/L.
7.	Consider potential causes of falsely low IGF1 (malnutrition, liver disease, inflammation) and GH peaks (obesity, acute stress).
